# An Onboard Vision-Based System for Autonomous Landing of a Low-Cost Quadrotor on a Novel Landing Pad

**DOI:** 10.3390/s19214703

**Published:** 2019-10-29

**Authors:** Xuancen Liu, Shifeng Zhang, Jiayi Tian, Longbin Liu

**Affiliations:** College of Aerospace Science and Engineering, National University of Defense Technology, Changsha 410073, China; liuxuancen14@nudt.edu.cn (X.L.); tianjiayi13@nudt.edu.cn (J.T.); liulongbin@nudt.edu.cn (L.L.)

**Keywords:** onboard vision, micro aerial vehicle (MAV), monocular camera, autonomous landing, low-cost sensors

## Abstract

In this paper, an onboard vision-based system for the autonomous landing of a low-cost quadrotor is presented. A novel landing pad with different optical markers sizes is carefully designed to be robustly recognized at different distances. To provide reliable pose information in a GPS (Global Positioning System)-denied environment, a vision algorithm for real-time landing pad recognition and pose estimation is implemented. The dynamic model of the quadrotor is established and a system scheme for autonomous landing control is presented. A series of autonomous flights have been successfully performed, and a video of the experiment is available online. The efficiency and accuracy of the presented vision-based system is demonstrated by using its position and attitude estimates as control inputs for the autonomous landing of a self-customized quadrotor.

## 1. Introduction

Over the past decade, significant progress has been achieved toward the automation of aerial robotic vehicles and related technology, leading to a variety of potential applications such as emergency response, traffic monitoring, inspection of power cables, package delivery, etc. [1,2]. micro aerial vehicles (MAVs) such as quadrotors are potentially able to move flexibly and explore efficiently in unforeseeable 3D environments with complex terrains, which is almost impossible for ground robots while executing time-sensitive missions. However, because of the limited payload and onboard battery endurance, micro quadrotors have a relatively shorter flight time than fixed-wing aircrafts, mostly ranging between 8 and 25 min. Consequently, micro quadrotors are programmed to land on a specific platform and get recharged periodically for operations that cover large areas. In such a scenario, how to precisely and robustly land an autonomous quadrotor on a ground platform is still a challenging task of MAVs, considering that the onboard processing power is strictly limited and sensors are micro.

As the most difficult and risky phase in flight, autonomous landing requires robust recognition of the landing platform and accurate measurement of the MAV’s movement with respect to the ground target. So far, it is still difficult to accurately estimate the relative position between the MAV and the landing platform unless using 3D light detection and ranging (Lidar) sensors or a differential global positioning system (DGPS), which are heavy and unaffordable [3,4,5]. For this reason, novel sensors are studied in many research works toward autonomous landing. [6] presented a low-cost visual tracking system for the hovering control of a Hummingbird quadrocopter by using a Wii remote infrared (IR) camera and a pattern of four infrared spots fixed on the landing pad. In [7], a low-cost solution based on a monocular camera was implemented for the autonomous takeoff, hovering, and landing of a MAV. By using projective geometry [8,9,10], the 6 degrees of freedom (DOF) pose of the quadrotor relative to a typical landing pad (the letter “H” surrounded by a circle) could be accurately estimated from image streams. In [11], a complete ship deck simulation for the autonomous landing of a helicopter on ships was proposed by using a single downward-looking camera and a moving platform with helipad marks. Another approach inspired by the behavior of insects used optical flow as feedback for MAVs in visual servo control [12], since it provided relative velocity to the dynamic environment [13]. Herissé et al. [14] presented a nonlinear controller for a vertical takeoff and landing (VTOL) unmanned aerial vehicle (UAV) that enabled hovering above and landing on a moving platform, by exploiting measurement of the average optical flow.

Compared with the sensors mentioned above, monocular cameras passively receive environmental information and have an inherent potential for object recognition tasks, while still being lightweight, low cost, and computationally efficient [15]. Unlike stereo cameras with limited baselines, monocular cameras are able to keep functioning even if the object is detected at a large distance. Considering that MAVs have limited payload and computational capability, monocular vision is comparably more attractive for autonomous landing and extended applications. In [16], AprilTag markers [17] were recognized by a single forward-looking camera mounted on the AR.Drone micro UAV, and state estimation was achieved based on a delayed-state extended Kalman filter (EKF) in GPS-denied situations. [18] presented an integrated visual detection system with a standard field of view camera lens and a fisheye lens occasionally used to capture faraway or highly close targets.

Due to the importance of autonomous localization and navigation, the issue of simultaneous localization and mapping (SLAM) has triggered a lot of interest in the research community, with the wide use of monocular vision. In [19], a model of the vision inertial absolute navigation system (VIANS) was established, and estimation of absolute navigation information was achieved based on the presented EKF and unscented Kalman filter (UKF). Shen et al. [20] and [21] presented a monocular visual–inertial system (VINS-Mono) for the 6DOF state estimation of UAVs, in which only a monocular camera and a low-cost inertial measurement unit were utilized. The proposed visual–inertial odometry (VIO) obtained high accuracy based on the real-time fusion of pre-integrated inertial measurement units (IMU) data and feature observations, and then the relocalization process started to eliminate accumulative drifts. In [22], a versatile visual marker-based multi-sensor fusion estimator was presented, which combined a variable optional number of sensors and positioning algorithms in a loosely coupling fashion. The results of estimation showed high accuracy in real experiments, controlling a quadrotor equipped with an IMU and an RGB camera.

In order to recognize the landing platform and precisely estimate the 6DOF pose with images captured by the onboard camera, artificial markers are widely used in autonomous landing tasks, especially square-based fiducial markers such as Matrix [23], ARTag [24], ARToolKit [25], ARToolKitPlus [26], AprilTag [17], and ArUco [27]. These markers are generally encoded by an inner binary code in order to be uniquely identified. With error detection and correction, the four corners of the identified marker can be used as reference points to estimate the camera pose by solving the Perspective-n-Point (PnP) problem [28,29,30,31,32,33], given that the camera is properly calibrated. Amongst these fiducial markers proposed in recent papers, ArUco markers, presented by Garrido-Jurado et al, have gained popularity in visual servo systems [34]. By using the ArUco library supported by OpenCV, it is not difficult to generate configurable dictionaries of markers and make a C++ program capable of identifying and localizing those markers within the predefined dictionary. However, of all the implemented marker-based autonomous systems, it is still a challenging task to acquire accurate pose estimates by using low-cost cameras with large image noises in the movement of a MAV. When getting much closer to the landing platform, the camera may lose part of the marker due to its restricted field of view (FOV) and rotation movement, so that the estimates of the camera pose will be extremely unreliable.

In this paper, motivated by the existing challenges mentioned above, an onboard monocular vision algorithm for the 6DOF pose estimation of a MAV is proposed by utilizing a consumer camera. A novel landing pad is designed with a predefined configuration of several ArUco markers surrounded by a circle, which observably improve the detection range and the robustness of recognition. With a dynamic weighed mean filter, a fusion estimation method is presented for a more accurate 6DOF pose with respect to the landing pad. Then, the relative position, velocity, and yaw angle are put into a cascade proportional-integral-derivative (PID) controller as input information to control the hovering flight of the MAV in GPS-denied situations, thus paving the way for autonomous landing on a specific landing pad. Based on the DJI F450 frame, an experimental quadrotor is self-customized and equipped with a low-cost camera at the price of $10, a microcontroller using consumer sensors, and an onboard computer for image processing. Compared with measurements of external positioning devices, the presented system is demonstrated to be reliable and cost-effective for the precision landing of an autonomous MAV.

The rest of this paper is organized as follows. In Section 2, the design of the landing pad and the algorithm used to identify the pattern are described. The estimation of the camera pose relative to the landing pad is presented and a method for fusion estimation is described. In Section 3, the dynamic model of the micro quadrotor is established, and the architecture of the overall landing system is presented. Section 4 describes the setup of the experiments, and practical results are given in this part. Finally, Section 5 concludes this paper.

## 2. Landing Pad Design and Vision Algorithm

### 2.1. Landing Pad Design and Recognition

The most commonly used landing pads—such as a letter “H” surrounded by a circle—are not highly featured and may not be uniquely identified in a cluttered environment. False positives caused by similar shapes of other objects in the background will inevitably reduce the robustness and reliability of the vision system. Compared with other patterns, square-based fiducial markers are an efficient approach to achieve both high speed and precision, which use an inner binary code for identification and error correction. The markers chosen for the design of the landing pad are generated from the predefined dictionaries of the ArUco library in OpenCV. Each marker is composed by a black border and an inner binary matrix (6 × 6 bits), which encodes its unique identification (id).

To detect and decode an ArUco marker, the image is converted to gray-scale and segmented using a local adaptive threshold strategy at the first step. Then, a contour detection and a polygonal approximation are performed. After the perspective projection is removed by computing the homography matrix, the resulting image can be divided into a grid, and each element is assigned a value of 0 or 1 by using the Otsu thresholding method [35]. Once the binary code is extracted, the marker and its unique identification will be determined if it actually belongs to the predefined dictionary.

**Figure 1:**
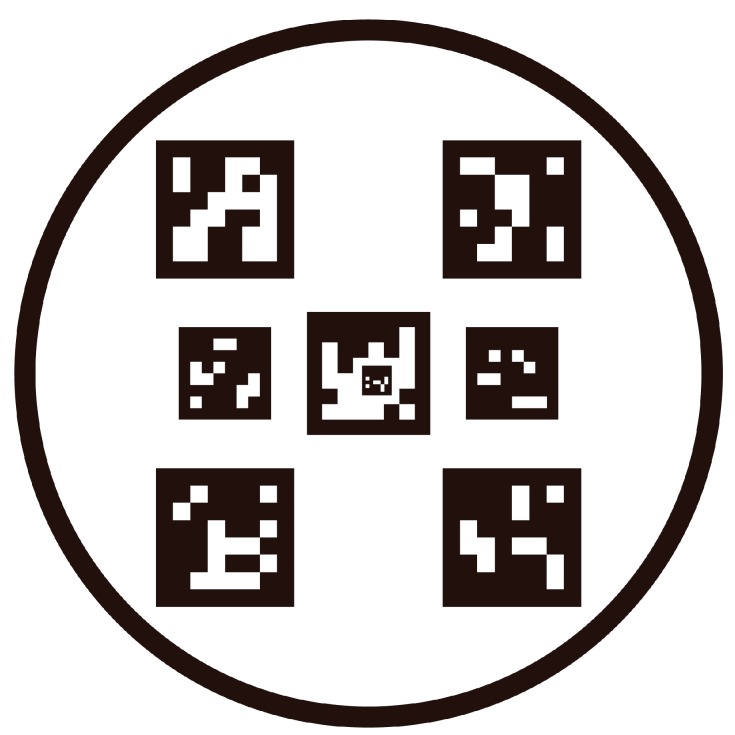
Configuration of the predefined landing pad.

In [36], the proposed Fractal Marker, a novel type of marker, is built as an aggregation of squared markers of different sizes, one into another, in a recursive manner, and a method for marker detection under severe occlusions is also presented. Taking into account the latest advances in the fractal ArUco, a novel landing pad with a wider detection range is designed in this paper to achieve a better performance in autonomous landing tasks. Figure 1 presents the predefined landing pad, which consists of eight unique ArUco markers surrounded by a circle. The radius of the circle is 700 mm, which facilitates its fast detection in the image at a large distance. The location of each marker is carefully designed, and the physical size of these markers ranges from the minimum (29 × 29 mm) to the maximum (135 × 135 mm), so that the desired marker can be kept within the restricted FOV of the onboard camera at different heights. It is worth mentioning that the smallest marker (29 × 29 mm) is placed inside the marker (120 × 120 mm) at the center of the landing pad, which is carefully designed so that the vision system will not lose all of the markers, even if the camera is very close to the landing pad. Based on this, a smaller “blind” range will be achieved.

**Figure 2:**
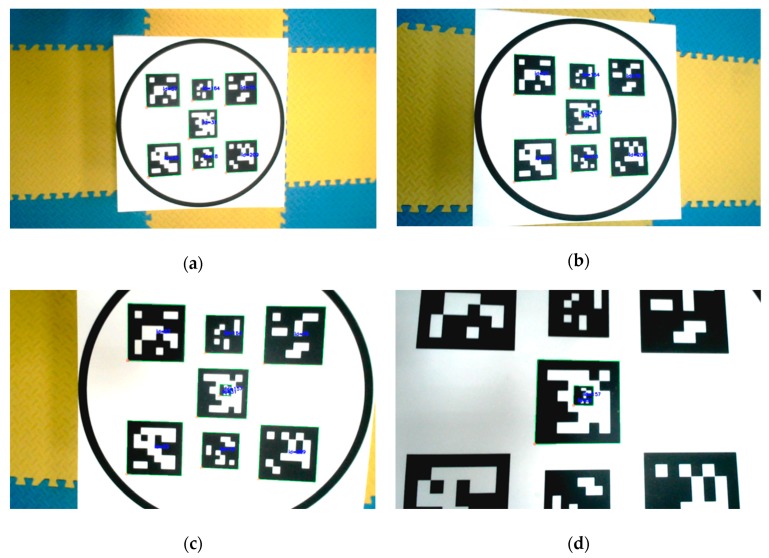
Markers recognized at different heights in camera view. When the camera is far away from the landing pad, the whole circle is captured in the view (**a**,**b**). With the change of attitude and height during a landing maneuver, part of the pattern moves out of the camera view (**c**). When the camera gets very close to the landing pad, only two markers can be recognized (**d**).

The optimal design and configuration of the landing pad significantly improves the accuracy of recognition instead of using an unaffordable camera with high resolution. As shown in Figure 2, the bigger markers can be detected and recognized at a larger distance, and if the UAV is much closer to the landing pad, the smaller ones could still be recognized.

To compute the relative position to the landing pad, the physical size and position of the detected marker must be known. When more than one marker is recognized, the measurement noise can be greatly reduced, and the precision can be improved by data fusion.

### 2.2. Relative Pose Estimation

The problem of solving the exterior orientation of a calibrated camera given reference 3D points and their corresponding 2D projections is commonly referred to as a Perspective-n-Point (PnP) problem, which is one of the most fundamental researches in computer vision. For large values of n, the direct linear transformation (DLT) method is commonly used to compute the camera pose. However, due to the overlook of the intrinsic camera parameters that we are able to acquire, the DLT method is comparatively inaccurate. The clamped DLT is an alternative method that exploits the known intrinsic parameters, but the accuracy is still low. Even though some non-iterative methods such as perspective-three-point (P3P) and efficient perspective-n-point (EPnP) are relatively fast at finding an optimal solution, these are not considered because they are not especially robust in planar cases, and sometimes lead to a mirror effect. Compared with these methods, the iterative method and the homography model for planar patterns are selected to estimate the camera pose, which is followed by nonlinear optimization using the Levenberg–Marquardt algorithm to minimize the reprojection error [37].

**Figure 3:**
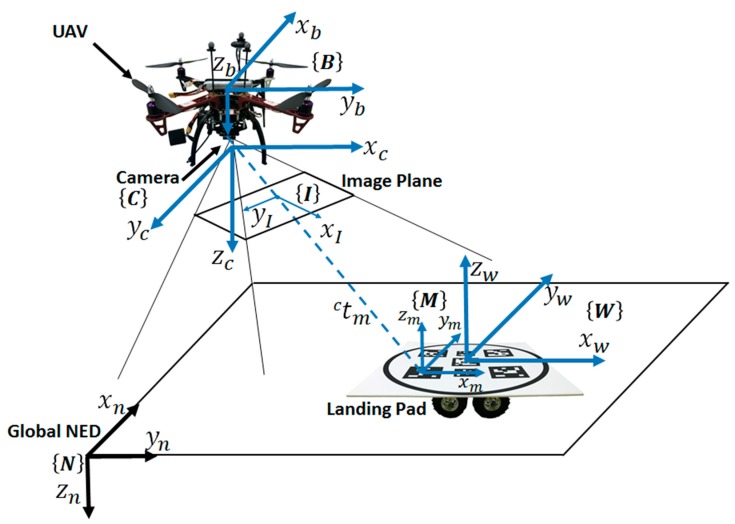
Definition of the unmanned aerial vehicle (UAV) body frame B, the camera frame C, the image frame I, the marker frame M, the world frame W, and a North–East–Down (NED) coordinate system taken as an inertial reference frame N.

The coordinate systems are defined in Figure 3. In the camera frame, the origin is the optic center, and $Z_{c}$ coincides with the optic axis of the camera. It is also worth mentioning that the origin of the world frame W is set at the center of the landing pad, which is attached to the ground platform.

Let Mmc be the homogeneous transformation matrix from the marker frame to the camera frame, which consists of a rotation matrix Rmc (3 × 3) and a translation vector tmc (3 × 1). The coordinate transformation from frame M to C is given as:

(1) [xcyczc1]=Mmc[xmymzm1]

where ${\left[x_{m},y_{m},z_{m}\right]}^{T}$ is the position of the reference point in the marker frame, ${\left[x_{c},y_{c},z_{c}\right]}^{T}$ is the corresponding position in the camera frame, and transformation matrix Mmc is:

(2) Mmc=[Rmctmc0T1]

In real flights, the camera frame rotates along with the quadrotor, while the $x_{m}y_{m}$ plane of the marker frame is kept horizontal. So, the inverse of Mmc will be more useful in this paper, which is given as:

(3) Mmc=Mm−1c=[Rm−1c−tmcRm−1c0T1]

where Mmc is the transformation matrix from the camera frame to the marker frame, and the pose of the quadrotor with respect to marker i can be estimated with analysis of it. As the landing pad is a cooperative target and the origin of the world coordinates is set at the center of the landing pad, the pose of marker i in the world frame is known. Thus, a translational transformation Mmw from the marker frame to the world frame can be calculated, which is given as:

(4) [xwywzw1]=Mmw[xmymzm1]

In the captured image, any point can be positioned by pixel values, which is generally defined as ${\left[u,v\right]}^{T}$. With a calibrated camera, the reference point can be transformed from the pixel values to the camera coordinates, which is given as:

(5) $$\left[\begin{array}{c} u\\ v\\ 1 \end{array}\right]=M_{in}\left[\begin{array}{c} x_{nc}\\ y_{nc}\\ 1 \end{array}\right]$$

where ${\left[x_{nc},y_{nc},1\right]}^{T}$ is the corresponding position on the normalized image plane, and $M_{in}$ is referred to as the intrinsic parameter matrix of the camera, which is expressed as:

(6) $$M_{in}=\left[\begin{array}{ccc} f/d_{x} & 0 & u_{0}\\ 0 & f/d_{y} & v_{0}\\ 0 & 0 & 1 \end{array}\right]$$

where f is the focal length, $d_{x}$ and $d_{y}$ are the physical length per pixel in the x and y axis directions, respectively, $\left(u_{0},v_{0}\right)$ is the intersection of the optical axis and the image plane in 2D pixel coordinates. As mentioned above, the landing pad and those planar patterns are located on the $z_{w}=0$ plane in W. Thus, the transformation from the normalized coordinates to the world coordinates can be expressed as:

(7) $$\left[\begin{array}{c} x_{nc}\\ y_{nc}\\ 1 \end{array}\right]=H\left[\begin{array}{c} x_{w}\\ y_{w}\\ 1 \end{array}\right]$$

where the 3×3 matrix H is a planar homography defined as:

(8) $$H=\left[\begin{array}{ccc} h_{11} & h_{12} & h_{13}\\ h_{21} & h_{22} & h_{23}\\ h_{31} & h_{32} & h_{33} \end{array}\right]$$

where $h_{ij}$ is the i,j-th element of H, which leads to equations as follows:

(9) $$\{\begin{array}{c} x_{nc}(h_{31}x_{w}+h_{32}y+h_{33})=h_{11}x_{w}+h_{12}y_{w}+h_{13}\\ y_{nc}(h_{31}x_{w}+h_{32}y+h_{33})=h_{21}x_{w}+h_{22}y_{w}+h_{23} \end{array}$$

When an ArUco marker is decoded and recognized, its four corners are located in the pixel coordinates and their corresponding position in the world frame can be derived from the predefined identification and the actual size of the detected marker. Given at least four such correspondences, a set of linear equations can be established to solve the elements of H:

(10) $$\left\{\begin{array}{ccccccccc} x_{w}^{1} & y_{w}^{1} & 1 & 0 & 0 & 0 & -x_{nc}^{1}x_{w}^{1} & -x_{nc}^{1}y_{w}^{1} & -x_{nc}^{1}\\ 0 & 0 & 0 & x_{w}^{1} & y_{w}^{1} & 1 & -y_{nc}^{1}x_{w}^{1} & -y_{nc}^{1}y_{w}^{1} & -y_{nc}^{1}\\ \vdots & \vdots & \vdots & \vdots & \vdots & \vdots & \vdots & \vdots & \vdots \\ x_{w}^{n} & y_{w}^{n} & 1 & 0 & 0 & 0 & -x_{nc}^{n}x_{w}^{n} & -x_{nc}^{n}y_{w}^{n} & -x_{nc}^{n}\\ 0 & 0 & 0 & x_{w}^{n} & y_{w}^{n} & 1 & -y_{nc}^{n}x_{w}^{n} & -y_{nc}^{n}y_{w}^{n} & -y_{nc}^{n} \end{array}\right\}h=0$$

where h is a 9×1 vector that contains all the $h_{ij}$ elements defined as $h={\left[\begin{array}{ccccccccc} h_{11} & h_{12} & h_{13} & h_{21} & h_{22} & h_{23} & h_{31} & h_{32} & h_{33} \end{array}\right]}^{T}$, ${\left[\begin{array}{ccc} x_{nc}^{i} & y_{nc}^{i} & 1 \end{array}\right]}^{T}$ is the i-th reference point in the normalized coordinate, and ${\left[\begin{array}{ccc} x_{w}^{i} & y_{w}^{i} & 1 \end{array}\right]}^{T}$ is the homogeneous coordinate in the world frame. Therefore, the matrix equation is simplified as:

(11) Ah=0

Thus, the solution can be computed by using known methods such as singular value decomposition (SVD) and the average reprojection error $R_{avg}^{E}$ can be minimized, which is defined as:

(12) $$R_{avg}^{E}=\frac{\sum_{i=1}^{n}\left\|x_{nc}^{i}-Hx_{w}^{i}\right\|}{n}$$

where n is the total number of corner correspondences.

### 2.3. Fusion Estimation

When more than one marker is recognized, pose estimates of different observed markers can be fused to form a better estimator. The previously estimated pose of the quadrotor with respect to the world frame given by every detected marker can be fused to get a more accurate result of the estimation. One way is to take a weighted average of the obtained estimates. Assuming that the estimator is unbiased, the variance of the original estimates should be taken into account to determine the weight coefficients. In general, the weights are chosen to minimize the variance of the weighted average such as a linear minimum variance estimator (LMVE).

Let $p_{i}$ be the original pose estimate of i-th detected marker, which is independent, and $\hat{p}$ be the unbiased estimate for the actual parameter p after taking the weighted average, which is as follows:

(13) $$\hat{p}=\omega_{1}p_{1}+\omega_{2}p_{2}+\cdots \omega_{n}p_{n}$$

where $\omega_{i}$($\sum_{i=1}^{n}\omega_{i}=1$ and $\omega_{i}\geq 0$ for all i) is the weight that is to be determined, and n is the total number of markers that are observed. The estimation error $\tilde{p}$ and known conditions are:

(14) $$\tilde{p}=p-\hat{p}$$

(15) $$E\left(\tilde{p}\right)=0$$

where $E\left(\tilde{p}\right)$ is the mathematical expectation; then, it can be proved by using the method of Lagrange multipliers that the variance is minimized when:

(16) $$\omega_{i}=\frac{\frac{1}{Var\left(p_{i}\right)}}{\sum_{j=1}^{n}\frac{1}{Var\left(p_{j}\right)}}$$

and its minimum value $MSE_{\min}$ is:

(17) $$MSE_{\min}=\frac{1}{\sum_{i=1}^{n}\frac{1}{Var\left(p_{i}\right)}}$$

While the quadrotor starts to descend and approach the landing pad, the weight $\omega_{i}$ is updated in real time according to the total number of markers that are recognized and the noise of the original data, thus leading to a more accurate and reliable result.

## 3. Dynamic Model and Landing System

### 3.1. Dynamic Model

The quadrotors are gradually emerging as a popular platform in aerial robotics research due to their low cost, the ability to hover, and the simplicity in mechanical structure. The vehicle consists of four brushless motors and four propellers providing the necessary force and moment for 6DOF motion control. Unlike helicopters that need complex mechanical control linkages, the quadrotors rely on four individual motors and the variation in speed to control the vehicle, which greatly simplifies the whole system.

**Figure 4:**
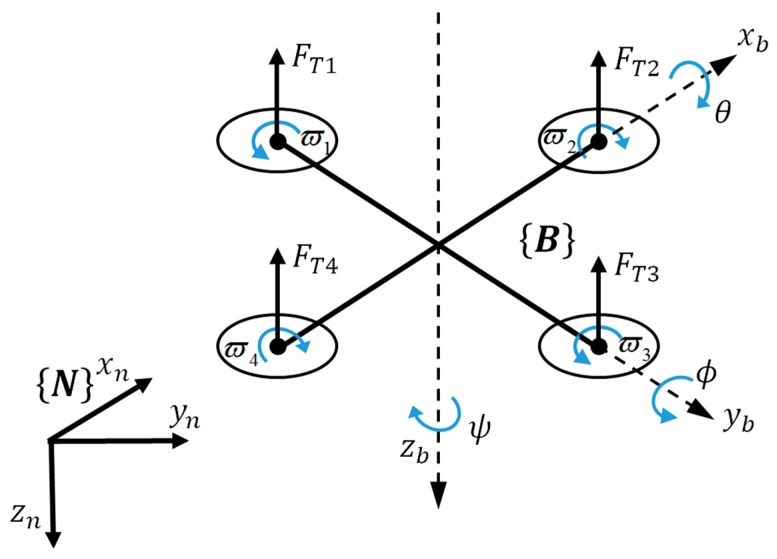
6DOF motion of a rigid quadrotor with corresponding frames.

The 6DOF motion of a rigid quadrotor and corresponding frames are described in Figure 4.

The quadrotor vehicle is represented by a rigid body of mass m and of moment of inertia J along with external forces and torques caused by propellers and gravity. A local North–East–Down (NED) frame N and a body fixed frame B attached to the UAV at the center of mass are introduced to describe the motion of the quadrotor. Let pn=[pxnpynpzn]T and vn=[vxnvynvzn]T be the position and linear velocity of the mass center relative to N. $\Theta ={\left[\begin{array}{ccc} \phi & \theta & \psi \end{array}\right]}^{T}$ is defined as the roll/pitch/yaw angles, which describes the orientation of the quadrotor in N. The rotation matrix $R_{b}^{n}$ from B to N is given as:

(18) $$R_{b}^{n}=\left[\begin{array}{ccc} \cos\theta \cos\psi & \sin\phi \sin\theta \cos\psi -\cos\phi \sin\psi & \cos\phi \sin\theta \cos\psi +\sin\phi \sin\psi \\ \cos\theta \sin\psi & \sin\phi \sin\theta \sin\psi +\cos\phi \cos\psi & \cos\phi \sin\theta \sin\psi -\sin\phi \cos\psi \\ -\sin\theta & \sin\phi \cos\theta & \cos\phi \cos\theta \end{array}\right]$$

The equations of motion for the quadrotor can be described as:

(19) p˙n=vn

(20) v˙n=gn3−fbmRbnn3

(21) J⋅ω˙b=−ωb×(J⋅ωb)+Ga+τ

where $f_{b}$ is the translational force applied to the quadrotor expressed in B, τ is the torque, g is the acceleration due to gravity, and ωb is the angular velocity of the MAV in frame B. The gyroscopic moment $G_{a}$, which is mainly generated by propellers, is not considered in this case. Furthermore, the translational dynamics shown in Equations (19) and (20) can be simplified as:

(22) p¨xn=−fbm(sinϕsinψ+cosϕsinθcosψ)

(23) p¨yn=−fbm(−sinϕcosψ+cosϕsinθsinψ)

(24) p¨zn=g−fbmcosϕcosθ

Furthermore, it can be assumed that sinϕ≈1, cosϕ≈1, sinθ≈θ, and cosθ≈1, considering that the roll and pitch angles are very small, which leads to a simplified dynamic model as described in [38].

### 3.2. Flight Control Algorithm

**Figure 5:**
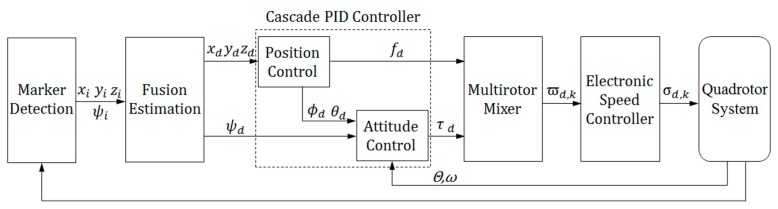
Hierarchical control architecture of a quadrotor.

Since the quadrotor is an underactuated system with four independent inputs, only the desired position ${\left[\begin{array}{ccc} x_{d} & y_{d} & z_{d} \end{array}\right]}^{T}$ and desired attitude $\psi_{d}$ can be directly tracked. Other variables such as $\phi_{d}$ and $\theta_{d}$ are determined by the known ones. The hierarchical control scheme of a quadrotor system is shown in Figure 5.

**Figure 6:**
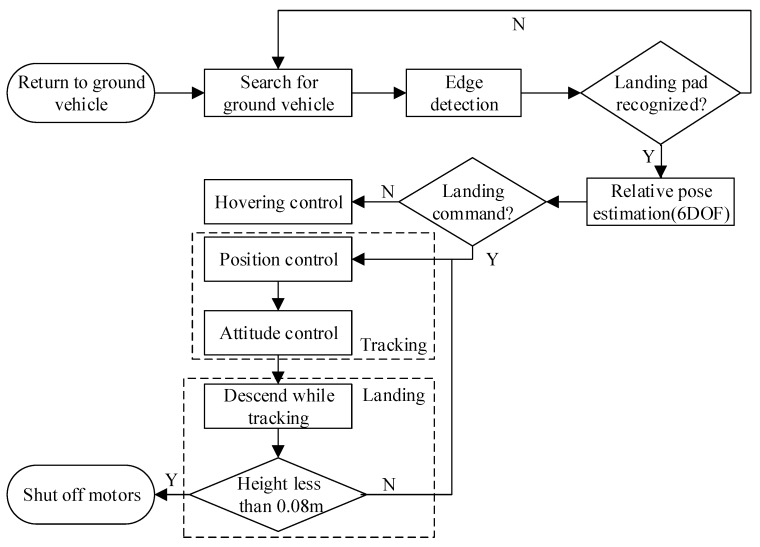
Overview of the proposed autonomous landing system.

The position ${\left[\begin{array}{ccc} x_{i} & y_{i} & z_{i} \end{array}\right]}^{T}$ and yaw angle $\psi_{i}$ of the quadrotor from marker i are estimated by the onboard vision system in the world frame, while the roll and pitch angles given by the onboard microcontroller using an EKF are expressed in global NED. The state of the quadrotor, the control desired position ${\left[\begin{array}{ccc} x_{d} & y_{d} & z_{d} \end{array}\right]}^{T}$, and the yaw angle $\psi_{d}$ are all expressed in the global NED. The position of the landing pad in global NED is exploited to calculate the state of the quadrotor. Cascade PID controllers are designed to individually control the 3D position and yaw angle of the quadrotor. The inner-loop controller for attitude control has been implemented on an open source autopilot. All PID gains have been preliminarily tuned in hovering flight tests. Considering that the thrust value is determined not only by the input of the desired position but also by the total takeoff weight of the quadrotor, the height controller is divided into two parts: a slightly changed base value for hovering and a fast controller for position control. An overview of the proposed landing system including landing pad recognition, 6DOF pose estimation, and flight control is shown in Figure 6.

When the landing pad is recognized by the onboard vision system, the quadrotor will maintain a constant descending velocity while continuing to track the target. With the landing pad mentioned above, the estimates obtained by the proposed system will not be divergent when the quadrotor is close to the target. Finally, due to the ground effect on the quadrotor, the motors are programmed to be shut off directly when the height is under 0.08 m and the horizontal distance must be less than 0.1 m simultaneously.

## 4. Experiments and Results

### 4.1. Experimental Setup

**Figure 7:**
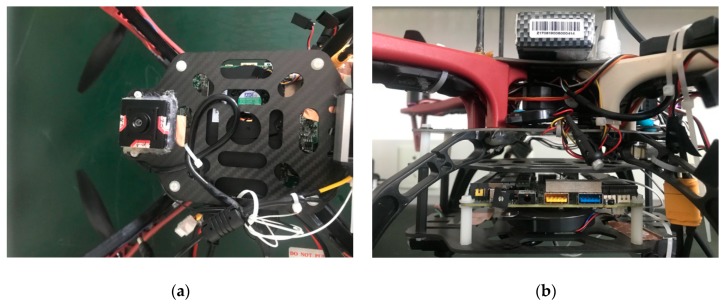
The low-cost camera (**a**) and onboard computer (**b**).

Most of the experimental UAVs are rather expensive with high-precision sensors and devices, which are unaffordable for practical applications. Instead of depending on high-resolution cameras that may cost more than $400, a low-cost consumer camera is used to establish the onboard vision system. The camera at a dimension of 35 × 35 × 30 mm^3^ weighs only 50 grams and has a maximum resolution of 1280 × 720 at 30 frames per second (fps), which makes it an ideal onboard sensor (see Figure 7a). With a constant focus length of 3.6 mm, the camera is aimed downward and attached to the bottom of the fuselage, covering a diagonal FOV of 90°. The price of only $10 is very attractive, considering its unexpected great performance. After calibration, the intrinsic parameters of the onboard camera are $f/d_{x}=912.5796$, $f/d_{y}=909.4341$, $u_{0}=669.2593$ and $v_{0}=322.0985$, while the distortion coefficient vector is ${\left[\begin{array}{ccccc} 0.0486 & -0.0907 & 0.0003 & -0.0009 & 0 \end{array}\right]}^{T}$.

**Figure 8:**
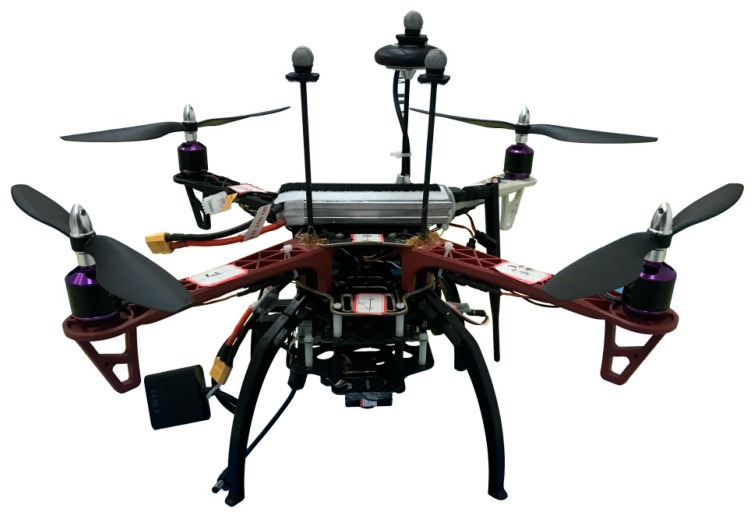
Overlook of the experimental platform in this paper.

As shown in Figure 8, the self-customized quadrotor is 45 cm in diameter based on the DJI F450 frame, and weighs 1.6 kilograms, including all the onboard devices and payloads. A 3300 mAh lithium battery powers all the motors and onboard electronics, which leads to a maximum flight time of up to 25 min.

**Figure 9:**
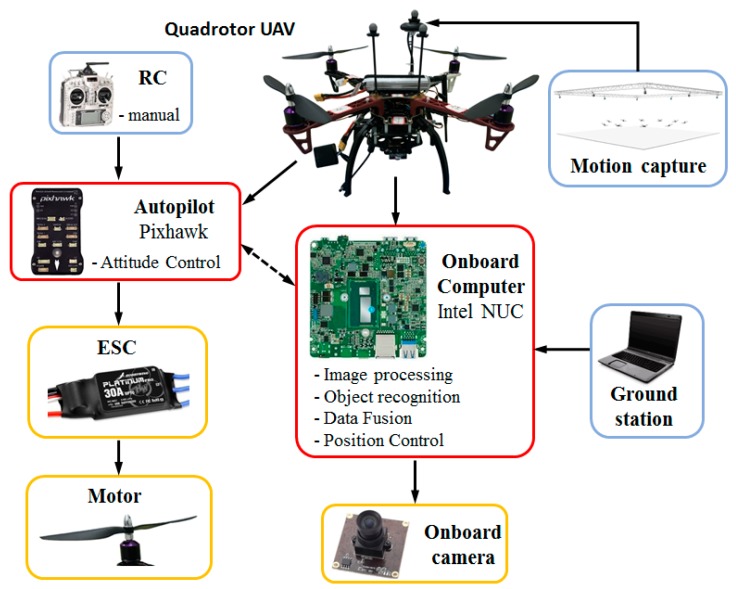
The architecture of the experimental system.

The overall system setup to perform the flight experiments for validation of the landing control algorithm is shown in Figure 9.

The inner-loop stabilization and attitude control of the quadrotor is achieved by utilizing a microcontroller developed by the Pixhawk team at ETU Zürich [39]. The Pixhawk controller is equipped with dual inertial measurement units (IMU) in case any one of them is malfunctioning. Many additional sensors and devices can be supported via drivers released by developer communities. The firmware version selected in this paper is 1.5.5.

**Figure 10:**
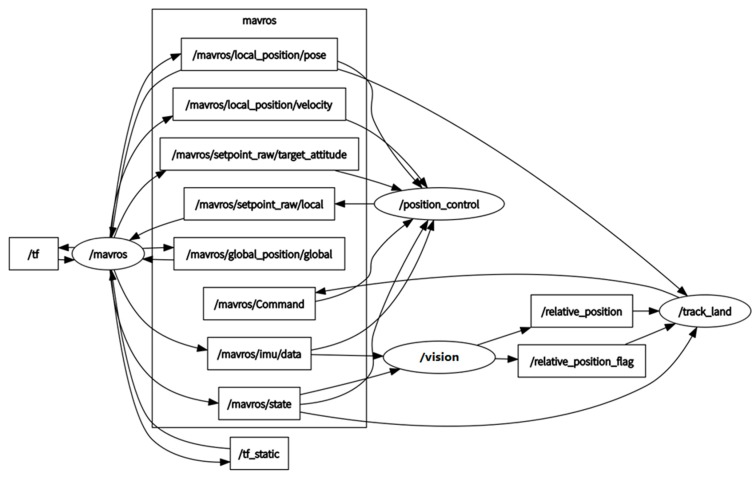
Robot Operation System (ROS) nodes and topics created to implement the algorithm.

In order to achieve more complex operations such as image processing, object recognition, data fusion, and position control, an Intel NUC with Core i5 processor is used as an onboard computer to perform the proposed vision algorithm. Based on serial communication, the current status and pose estimates can be transferred from the NUC to the Pixhawk flight controller as input information, which is developed based on a MAVLINK [40] extendable communication node for the Robot Operation System (ROS) [41], as shown in Figure 10. A higher estimation and control rate will significantly improve the accuracy, if supported by the hardware. However, in this paper, the estimation rate is at 25 Hz, and is limited by the onboard computation power. Thus, the control rate cannot be faster than 25 Hz under the current conditions. By using a 2.4 GHz remote controller (RC), the quadrotor can be controlled manually at the beginning of the experiment, and then switched to the offboard mode triggered by an RC signal.

ROS nodes are basic executable programs that process information to communicate with other nodes. In this system, the necessary nodes and topics shown in Figure 10 have been created and used to achieve the vision-based autonomous landing algorithm. Predefined topics can be published or subscribed by these nodes, thus passing the messages from one to another.

Once the landing procedure is launched, the quadrotor is in a fully autonomous mode, and only the onboard images along with IMU data can be utilized to provide navigation information. Since no GPS data can be received in indoor environments, a motion capture system (OptiTrack [42]) is used to provide the 6DOF pose of the quadrotor in real time at 100 Hz as the ground truth data, which can be compared with the vision system. To evaluate the proposed system, pose estimates of each detected marker have also been recorded as a reference value.

**Figure 11:**
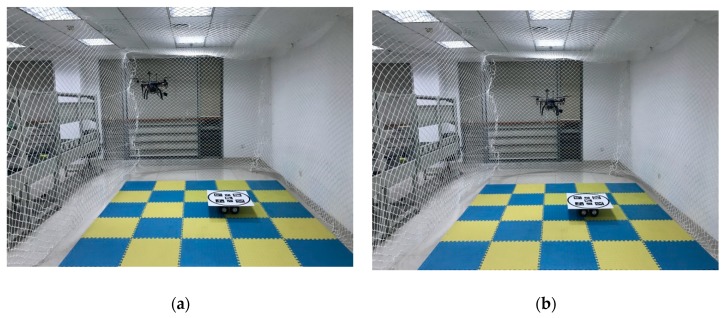

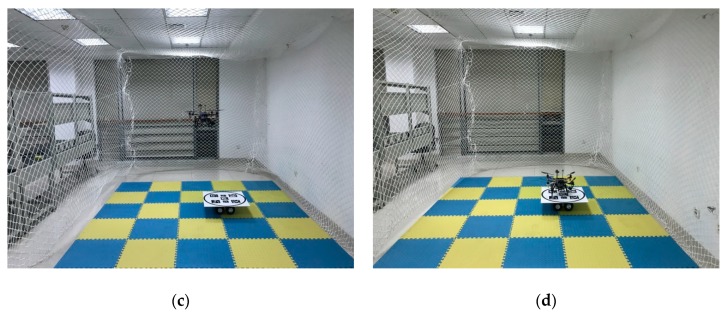
Autonomously approaching (**a,b**), descending (**c**), and landing on a static landing pad (**d**).

An autonomous landing test is shown in Figure 11.

### 4.2. Hovering Flight Control and Accuracy Analysis

During the indoor experiments, the GPS and air pressure sensor for position control were disabled. The desired yaw angle was set to be zero degrees. Under such conditions, a series of hovering and hand-held tests at different heights have been conducted with the onboard vision system. Compared with hand-held cases, the vibration of the quadrotor in real flight could increase errors to the pose estimates, but the actual impact was not so great in hovering flight. This is not a surprise, because the roll and pitch angles are quite small, which will not introduce large deviations. By comparing to the ground truth data, the root mean square errors (RMSEs) of the proposed vision algorithm during the whole flight are listed in Table 1 (in the Estimated row). As a contrast, the data without fusion estimation is listed (in the Single marker row).

**Figure 12:**
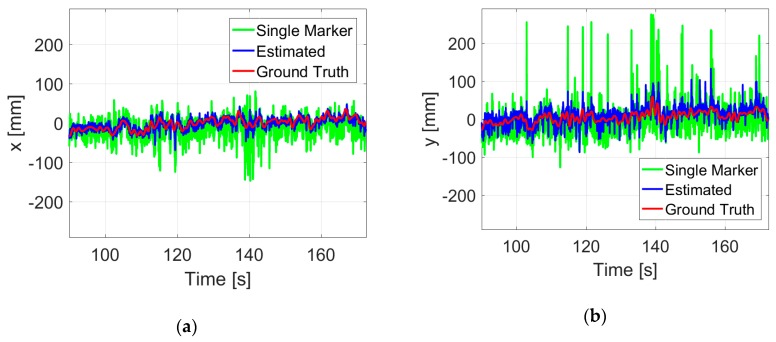

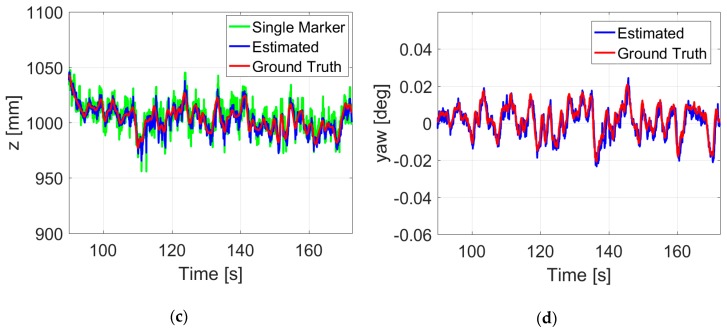
Position (**a**–**c**) and yaw angle (**d**) estimates during a hovering flight at 1 m height.

In about 82 s flight at 1 m height, an accuracy of ±10.1 mm in the x position and ±19.1 mm in the y position could be achieved, which is much better than that of a single ArUco marker. The deviation of 3D position estimates is 22.2 mm, thus leading to an accuracy of 23.2 mm in hovering control measured by the motion capture system. Figure 12 shows a more detailed record of the flight. Results of the proposed vision system are plotted in blue, the single marker is plotted in green, and the ground truth data are plotted in red.

It is obvious that the proposed estimates plotted in blue are well in line with the ground truth data throughout the flight test. Outliers caused by image noises have been significantly filtered by the proposed fusion estimation method. With the low-cost onboard vision system, high-precision control of autonomous hovering flight is achieved.

### 4.3. Autonomous Landing

**Figure 13:**
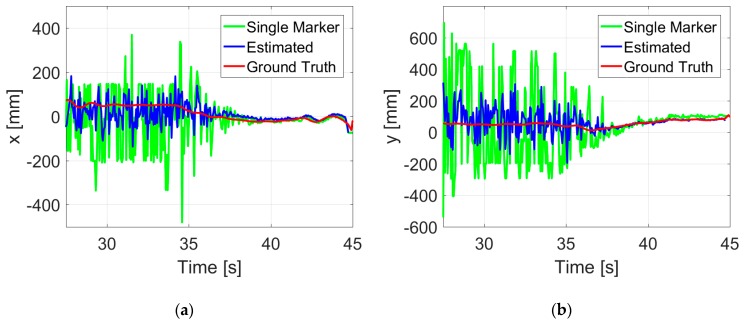

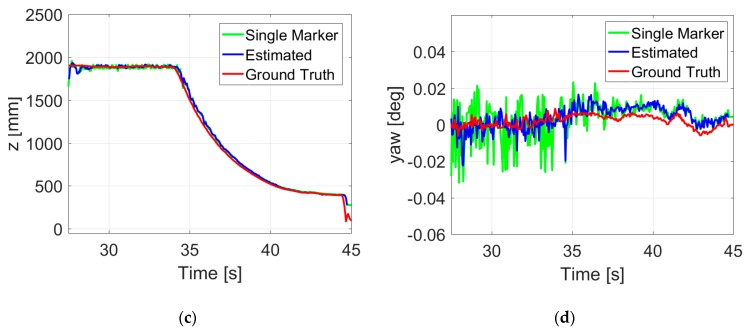
Position (**a**–**c**) and yaw angle (**d**) estimates during an autonomous landing.

This section presents the results of autonomous landing on the static landing pad, which is fixed on a ground platform. Once the quadrotor is armed and switched to the offboard mode, it will take off autonomously to the set point and keep hovering until the next command is received. As shown in Figure 13, after 7 seconds of hovering around the set point ${\left(0,0,1900\right)}^{T}$ (mm), the quadrotor received a command to land, and it took about 8 s to descend. Then, the onboard system started to determine whether the shut-off condition was met or not. The process was repeated until the destination area was reached, where the quadrotor could be blindly powering down. The final position of the quadrotor is very close to the center of the landing pad, which reflects the significance of this novel landing pad. The results are plotted in the world frame to better show the relative pose of the quadrotor with respect to the landing pad.

**Figure 14:**
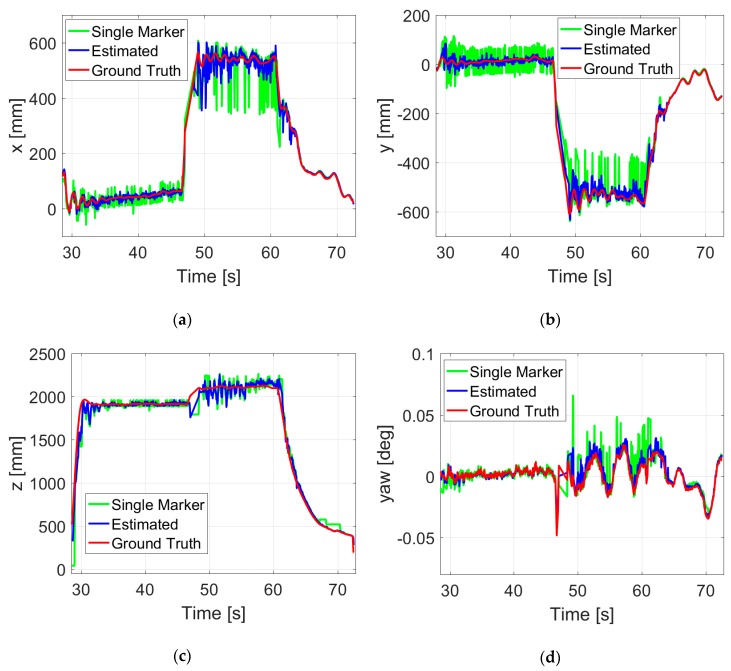
Position (**a**–**c**) and yaw angle (**d**) estimates during an indoor autonomous takeoff, hovering, and landing flight.

Figure 14 shows the pose estimates in a successful takeoff, hovering, moving, and landing flight. For the beginning of the takeoff phase, the quadrotor ascended without closed-loop control until one of the markers was recognized. A command made by the ground station was sent to the onboard system, and then the quadrotor moved to the set point ${\left(550,-550,2100\right)}^{T}$ (mm). After hovering about 10 s, it started the landing phase, and did not take much time to meet the shut-off condition.

Some oscillations still remain in the current configuration, which occurred with sudden changes of the quadrotor’s attitude. In this paper, the camera is fixed to the bottom of the quadrotor, and so it rotates along with the body frame. The estimation error will significantly increase when the orientation of the camera changes quickly in a very short time, especially at a relatively low estimation rate. A higher operating frequency would significantly improve the stabilization, but is prevented by the limited ability of onboard image processing. In fact, considering the cost of all the sensors and devices, the performance of the proposed vision system is quite attractive in practical applications.

A video of these experiments demonstrating autonomous flights with the proposed system is available at Supplementary Materials section.

## 5. Conclusions

In this paper, we have presented an onboard vision system that consists of a downward-looking camera, a microcontroller, and an image processor to visually and autonomously provide position and attitude estimates of the MAV with respect to the ground platform. A novel landing pad using different ArUco marker sizes is carefully designed for autonomous landing tasks, ensuring the detectability at different distances. With the proposed algorithm, position and attitude estimates of the MAV can be calculated by utilizing reference points extracted from the pad image. A method of fusion estimation has proved its effectiveness in experiments.

We demonstrated the proposed vision algorithm through a series of experiments, which enabled a self-customized quadrotor to autonomously take off from, hover above, track, and land on a static landing pad. Evaluated by an external tracking system, the results of real-time flight experiments have shown the feasibility, robustness, and accuracy of the vision algorithm. Considering that the cost of sensors is very low, the achieved accuracy is very attractive and sufficient for autonomous landing tasks.

There are several directions in which future work is of interest. To achieve more robust and accurate measurements, the sensor fusion of both IMU data and the vision system is worth investigating. Extra sensors such as infrared camera and Lidar may be used to expand the application of MAVs. Furthermore, we plan to place the camera in a gimbaled platform, which enables the MAV to track and approach a moving target.

## Figures and Tables

**Table 1:** Root mean square errors (RMSEs) of hovering flight at 1 m in different cases.

RMSEs (mm)	x	y	z	xy Plane	3D
Single marker	30.8	44.0	8.9	53.7	54.4
Estimated	10.1	19.1	5.5	21.6	22.2

## Supplementary Materials

The following are available online at https://www.mdpi.com/1424-8220/19/21/4703/s1, Video S1: Onboard Monocular Vision System for Autonomous Landing of a Low-Cost MAV in GPS-Denied Environment.
